# Ingested house dust mite favors sensitization to egg white in mice independently of its proteinase activity

**DOI:** 10.3389/fimmu.2024.1505003

**Published:** 2025-01-20

**Authors:** Sara Benedé, Leticia Pérez-Rodríguez, David Menchén-Martínez, Elena Molina, Rosina López-Fandiño

**Affiliations:** Instituto de Investigación en Ciencias de la Alimentación (CIAL), CSIC-UAM, Madrid, Spain

**Keywords:** food allergy, house dust mite, proteinase activity, egg white, oral sensitization

## Abstract

**Background:**

It is well-documented that house dust mite (HDM) exposure can cause tissue damage and activate innate immune responses. However, its role in promoting gastrointestinal sensitization and allergenicity to food proteins has been relatively unexplored.

**Methods:**

This study investigates the immunostimulatory effects of HDM in a murine model of oral sensitization to egg white (EW) in the absence of exogenous adjuvants. Additionally, we examined a proteolytically inactivated form of HDM (iHDM) to assess the contribution of HDM protease activity to its adjuvant potential.

**Results:**

Both HDM and iHDM enhanced allergic responses to EW proteins via the oral route, evidenced by mast cell degranulation in the intestinal tract upon EW challenge. Notably, only iHDM induced detectable concentrations of serum EW-specific IgE and IgG1 antibodies. Whereas HDM increased intestinal expression of genes encoding tight junction proteins and Th2-inducing alarmins to a greater extent than iHDM, active proteinases were not required for its adjuvant activity, as iHDM preferentially promoted Th2 responses in intestinal lymphoid tissues.

**Conclusions:**

These findings suggest that ingestion of environmental dust may contribute to food allergy development and highlight the complex and context-dependent nature of the adjuvant activity of HDM.

## 1 Introduction

Damage to the intestinal epithelial barrier is considered a contributing factor to the multifaceted etiology of food allergy and the rising prevalence and severity of this condition (1). In this context, certain functional features of food allergens, such as their proteolytic activity, have been associated with their role in the initiation and development of allergic responses through the disruption of the intestinal function (2). Whereas only some food allergens are proteinases, proteolytic enzymes are prevalent in major airborne allergens, such as house dust mite (HDM) or fungi (3). Notably, the immunostimulatory properties of HDM have been identified as a risk factor, leading to sensitization through non-oral routes, such as the airways, to inhaled food allergens (4). In addition, cases of anaphylaxis due to ingestion of HDM-contaminated food have also been reported (5, 6). Furthermore, major HDM allergens, including Der p 1, Der p 2, and Der p 23, have been detected in the gastrointestinal tract of human subjects (7, 8).

Cysteine proteases (Der p 1) and serine proteases (Der p 3, Der p 6, and Der p 9) are considered responsible for the remarkable sensitizing potential of HDM (9). In the respiratory tract, HDM proteases can increase airway epithelial permeability by degrading tight junction proteins that maintain barrier function and regulate paracellular transport, either directly or indirectly through the activation of protease-activated receptors. The result is the release of Th2-inducing alarmins interleukin (IL) 33, IL-25, and thymic stromal lymphopoietin (TSLP) and proinflammatory cytokines (IL-6, IL-1β, etc.), which stimulate innate immune mechanisms, mainly involving dendritic cells (DCs) and group 2 innate lymphoid cells (ILC2s), ultimately driving the differentiation of Th2 cells and the production of allergen-specific Immunoglobulin (Ig) E (2, 10). In addition to this mechanism, Toll-like receptor (TLR) signaling, particularly TLR4 signaling, is an important element in the development of airway sensitization to HDM allergens (11). Der p 1 allergens rely on their proteolytic activity, which leads to matured thrombin that cleaves fibrinogen, to activate TLR4 (12), whereas non-proteolytic HDM allergens, such as Der p 2, interact with TLR2 and TLR4 through lipids bound to their hydrophobic pockets (13).

These observations suggest that, given that HDM can cause tissue damage and activate innate immune cells, it may also have the potential to promote sensitization and enhance allergenicity to food proteins through the gastrointestinal tract, an aspect that has been relatively unexplored (14). In a previous study, we found that HDM extracts, orally administered to mice during a short period, exert protease-dependent and independent effects on innate immunity in the intestine through the upregulation of genes encoding proinflammatory and Th2-biasing alarmins and the activation of DCs and ILC2s (15). However, the experimental setting used did not allow for the demonstration of whether HDM could promote sensitization to bystander food proteins, as this would require a long-term oral administration model to generate adaptive immunity.

In this study, we used a murine model of oral sensitization, without exogenous adjuvant, to investigate the immunostimulant properties of HDM in the development of allergy to egg white (EW). With the aim to evaluate the contribution of protease activity to its potential adjuvant effect, both the proteolytically active and inactive forms of HDM were separately assessed.

## 2 Materials and methods

### 2.1 Samples

An HDM extract, low in endotoxin and with a known content of Der p 1 (10.2 µg mg^−1^ dry weight), was purchased from Citeq Biologics (Groningen, The Netherlands). The proteolytically inactive extract (iHDM) was obtained by treatment with the serine protease inhibitor 4-(2-aminoethyl) benzenesulfonyl fluoride hydrochloride and the cysteine protease inhibitor E-64 (both from Sigma-Aldrich, San Luis, Missouri, USA), at concentrations of 100 mM and 10 mM, respectively, at 37°C for 30 min. Excess of inhibitors was removed using PD-10 desalting columns (GE Healthcare, Chicago, Illinois, USA). Its proteolytically active counterpart (HDM) was mock-treated by using a similar desalting procedure, although it was not previously incubated with protease inhibitors (15). Protein concentration was determined using the bicinchoninic acid assay (Thermo Fisher Scientific, Waltham, Massachusetts, USA), following the manufacturer’s instructions. The protease activity of the proteolytically active and inactive extracts was assessed using the fluorogenic peptide substrate Boc-Gln-Ala-Arg-AMC. This confirmed that treatment of the extract with serine and cysteine protease inhibitors abolished its proteolytic activity (**Supplementary Figure S1**). EW was carefully separated from fresh eggs and lyophilized. Its protein content, lipid composition, and absence of cross-contamination were examined as previously indicated (16). The absence of lipopolysaccharide (LPS) in EW, HDM, and iHDM was confirmed by using the transfected cell line THP1-XBlue™, stably expressing an Nuclear factor kB/activator protein 1 (NF-kB/AP-1)–inducible secreted alkaline phosphatase reporter, as assessed by the QUANTI-Blue™ assay (InvitroGen, Carlsbad, CA, USA), following the manufacturer’s instructions. This indicated levels of LPS lower than 1 EU µg^−1^ in all cases.

### 2.2 Experiments in mice

**Figure 1:**
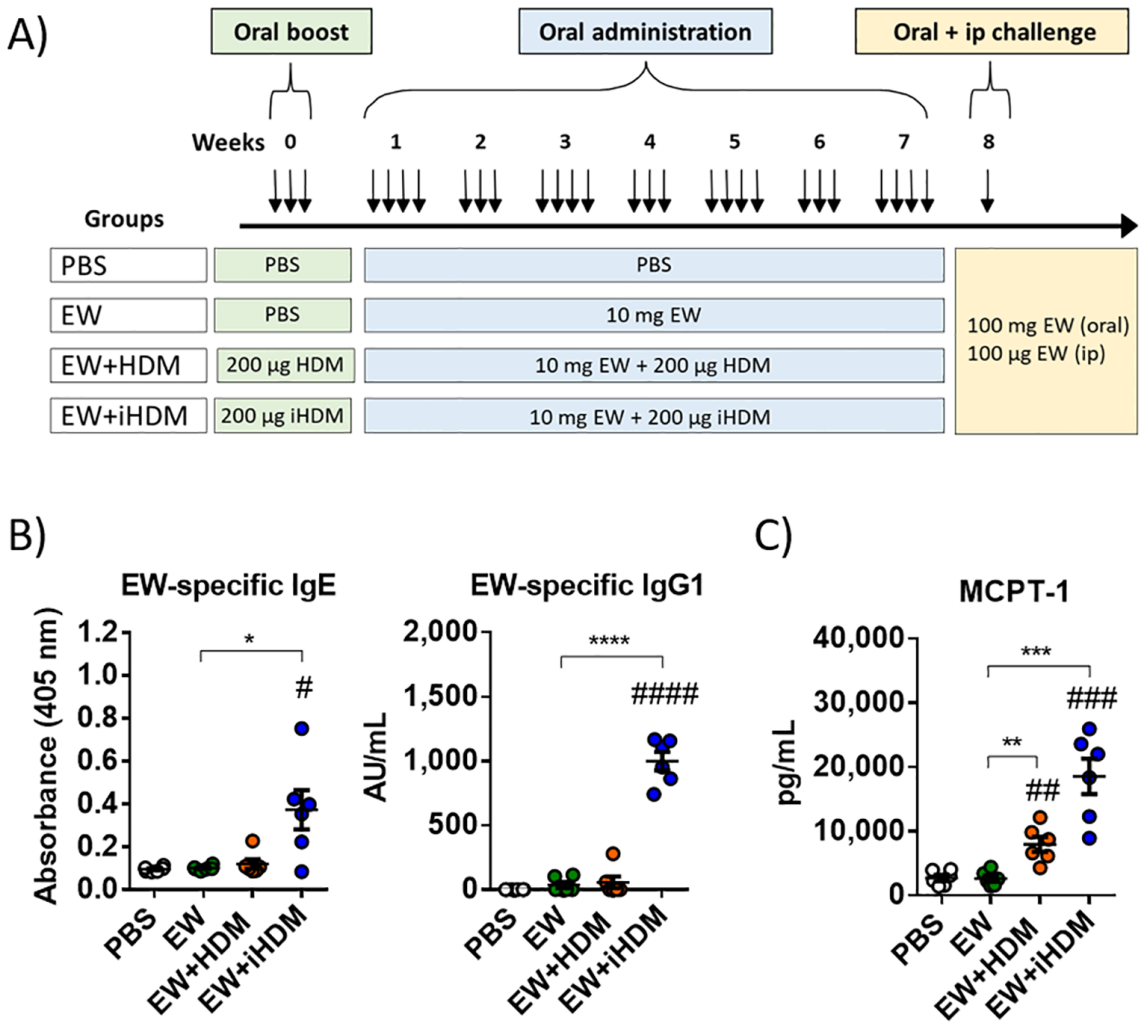
**(A)** Experimental protocol in animals. **(B)** Serum EW-specific IgE and IgG1. **(C)** Serum mast cell proteinase-1 (MCPT-1). Data are expressed as means ± SEM (*n* = 6). Pounds and asterisks indicate, respectively, statistically significant differences with respect to mice administered PBS orally or among the various experimental groups. # and * *p* < 0.05; ## and ** *p* < 0.01; ### and *** *p* < 0.001; #### and **** *p* < 0.0001.

Six-week-old female BALB/c mice were purchased from Charles River Laboratories (Saint Germain sur l’Arbresle, France). All protocols followed the European Legislation (directive 2010/63/EU) and were approved by the Comunidad de Madrid (Ref PROEX 286.8/20). For oral sensitization experiments, mice (*n* = 6 per group) received, intragastrically, 200 µg of HDM or iHDM for three consecutive days, followed, respectively, by combinations of EW + HDM and EW + iHDM, in a proportion of 10 mg + 200 µg, every other day for 7 weeks (17, 18). The doses of EW and mite extracts were chosen according to a previous acute administration study and they were administered with an interval of 30 min to avoid affecting the proteolytic activity of HDM by egg protease inhibitors or causing early degradation of EW proteins (15). Two groups of mice, receiving PBS orally (200 µL) for 3 days followed by either PBS or EW (10 mg) for 7 weeks, were used as controls. Twenty-four hours after the last administration, mice were orally challenged with 100 mg of EW, followed, 40 min apart, by an intraperitoneal challenge with 100 μg of EW (**Figure 1A**).

Body temperature and anaphylactic responses were evaluated 30 min after each challenge and mice were euthanized by CO_2_ inhalation 30 min later (19). Serum EW-specific IgG1 and IgE were analyzed by indirect Enzyme-linked immunoSorbent assay (ELISA) and capture ELISA, respectively (20), and mast cell protease 1 (MCPT-1) was determined with an ELISA Kit (eBioscience, San Diego, USA). Jejunum and mesenteric lymph nodes (MLNs) were individually recovered (18), and gene expression analyses were performed as described in **Supplementary Table S1**. Cytokines were quantified with ELISA kits (InvitroGen) in the supernatants of jejunum samples homogenized in PBS (21). ILC2s and DCs from the lamina propria (LP) were isolated and analyzed by flow cytometry (18). MLN cells were stimulated with EW (50 μg mL^−1^) and, 2 h later, with Brefeldin A (1 μg mL^−1^; BD Biosciences, San Diego, CA, USA) for 14 h, before T cells were analyzed by flow cytometry (18).

### 2.3 Statistical analyses

Results are presented as means ± SEM except for clinical signs, which are expressed as medians. Differences were determined by one-way or two-way ANOVA, followed by Tukey *post-hoc* test, except for gene expression data and clinical sign scores, for which the Mann–Whitney U test was used. p < 0.05 was considered statistically significant. Statistical analyses were performed using GraphPad Prism v6 (GraphPad Software Inc., San Diego, CA).

## 3 Results

BALB/c mice were orally exposed, without the addition of exogenous adjuvants, to HDM or iHDM for 3 consecutive days, followed by the administration of EW combined with HDM or iHDM every other day for 7 weeks, before an oral challenge with a high dose of EW and a subsequent systemic (intraperitoneal) challenge (**Figure 1A**). Only the mice that received the mixture of EW and iHDM produced EW-specific IgE and IgG1 antibodies (**Figure 1B**). Neither oral nor intraperitoneal challenge with EW elicited clinical symptoms or changes in body temperature in any of the experimental groups (not shown). However, the serum concentration of MCPT-1 was significantly increased in mice receiving EW + HDM and, particularly, in those receiving EW + iHDM (**Figure 1C**), indicating that both extracts induced a mast cell response to EW, although only the inactive extract facilitated the development of detectable levels of EW-specific Th2-driven antibodies.

**Figure 2:**
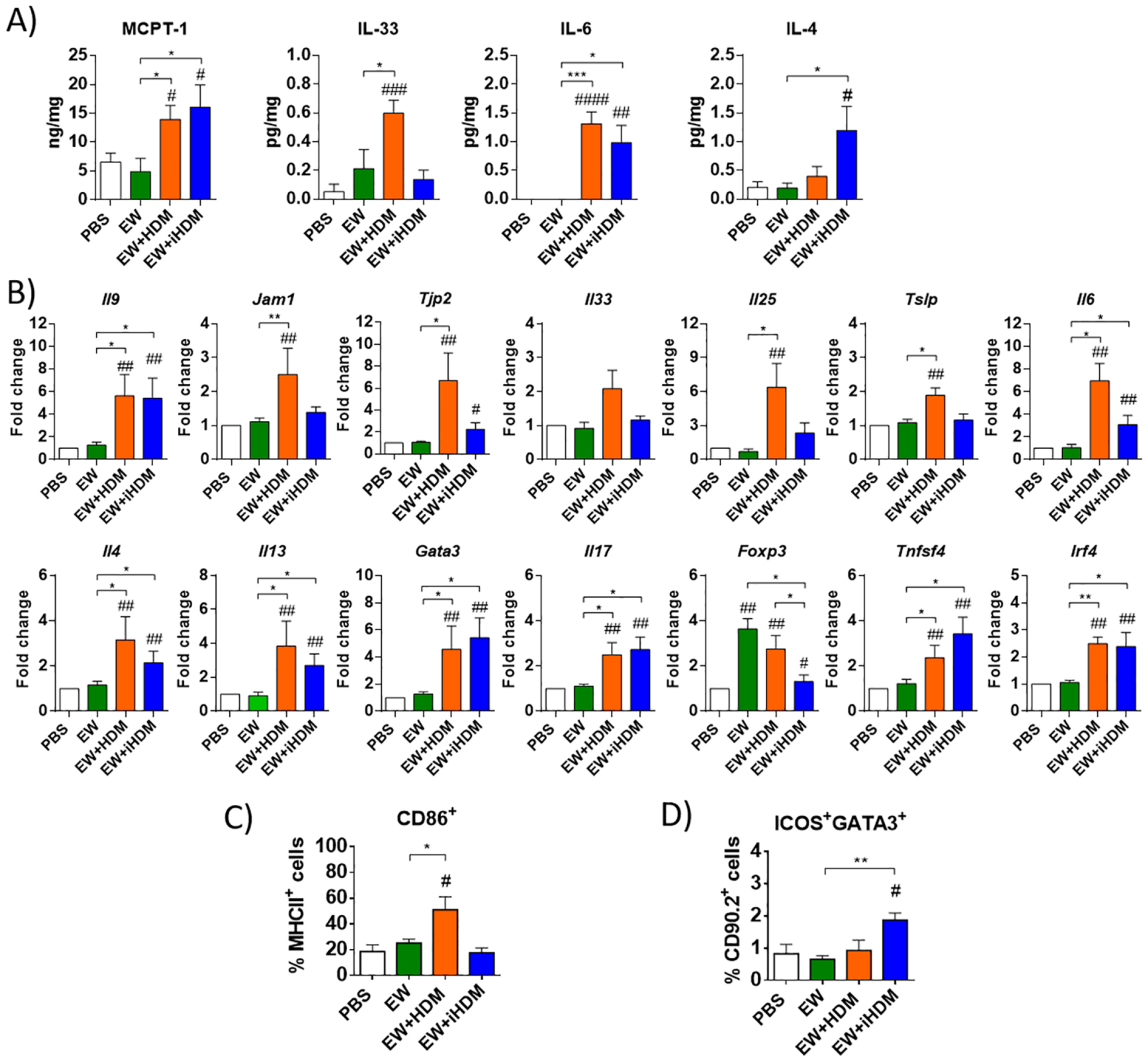
**(A)** Concentration of MCPT-1, IL-33, IL-6, and IL-4 in jejunum homogenates. **(B)** Relative gene expression of *Il9* (IL-9), *Jam1* (junctional adhesion molecule 1), *Tjp2* (zonula occludens 2), *Il33* (IL-33), *Il25* (IL-25), *Tslp* (TSLP), *Il6* (IL-6), *Il4* (IL-4), *Il13* (IL-13), *Gata3* (GATA3), *Il17* (IL-17), *Foxp3* (Foxp3), *Tnfsf4* (OX40L), and *Irf4* (IRF4) in the jejunum of mice. **(C)** Expression of CD86 in lamina propria dendritic cells (defined as CD11c^+^ cells expressing MHCII). **(D)** Group 2 innate lymphoid cells [defined as GATA3^+^ICOS^+^ within the CD90.2^+^ population containing CD45.2^+^ Lineage^−^ (CD3^−^ CD45R^−^ CD11b^−^ TER-119^−^ Ly-G6^−^ CD19^−^)] in the lamina propria of mice. Gene expression was normalized to the reference gene *Actb* and compared with mice administered PBS. Data are expressed as means ± SEM (*n* = 6). Pounds and asterisks indicate, respectively, statistically significant differences with respect to mice administered PBS orally or among the various experimental groups. # and * *p* < 0.05; ## and ** *p* < 0.01; ### and *** *p* < 0.001; #### p < 0.0001.

The jejunal concentration of MCPT-1 (**Figure 2A**) and the expression of *Il9* (**Figure 2B**) were significantly increased in mice orally exposed to EW + HDM and EW + iHDM, also pointing to degranulation of intestinal mast cells upon challenge with EW. Administration of EW + HDM upregulated the expression of *Jam1* in the jejunum and both EW + HDM and EW + iHDM enhanced that of *Tjp2* (**Figure 2B**), whereas the expression of *Cldn2*, *Cldn3*, and *Tjp1* remained unchanged (not shown). Oral exposure to EW + HDM increased the expression of *Il25*, *Tslp*, and *Il6* and the jejunal concentration of IL-33 and IL-6, whereas that of EW + iHDM led to high levels of IL-6 and IL-4 (**Figure 2A**). The enhanced upregulation of genes encoding tight junction proteins and Th2-inducing alarmins suggests that HDM caused damage to the intestinal epithelium due to its proteolytic activity. Indeed, the administration of the proteolytically active extract promoted DC maturation in the LP, as indicated by increased levels of CD86 expressing CD11c^+^MCHII^+^ cells (**Figure 2C**), although the examination of the gene expression of DC Th2-biasing factors (*Tnfsf4* and *Irf4*) revealed a similar pattern between mice exposed to EW + HDM and EW + iHDM (**Figure 2B**). Nevertheless, notably, the administration of EW + iHDM increased the numbers of ILC2s in the LP (**Figure 2D**). Both EW + HDM and EW + iHDM upregulated *Il4*, *Il13*, *Gata3*, *Il17*, and *Foxp3* in the intestine (**Figure 2B**). Of note, the jejunal expression of *Foxp3* [encoding forkhead box P3 (Foxp3), a master regulator of regulatory Tcells] in mice that received EW and EW + HDM than that in mice from the EW + iHDM group.

**Figure 3:**
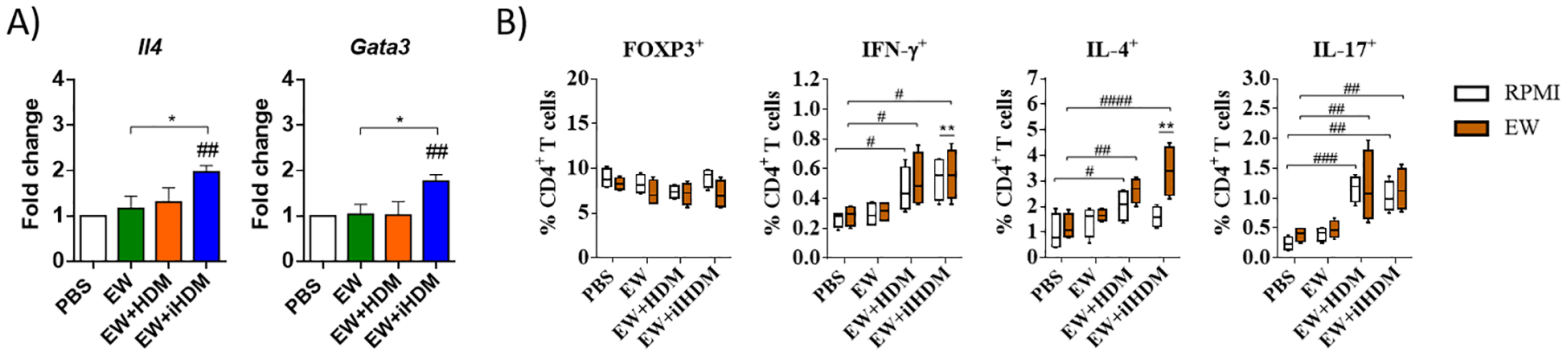
**(A)** Relative gene expression of *Il4* (IL-4) and *Gata3* (GATA3) in the mesenteric lymph nodes of mice. **(B)** Mesenteric lymph node T cells (defined as CD3^+^CD4^+^ cells) expressing intracellularly FOXP3 or the cytokines IFN-γ, IL-4, and IL-17 after culture with EW. Data are expressed as means ± SEM (*n* = 6). Pounds and asterisks indicate, respectively, statistically significant differences with respect to mice administered PBS orally or among the various experimental groups. # and * p < 0.05; ## and ** p < 0.01; ### p < 0.001; #### p < 0.0001.

Regarding intestinal lymphoid organs, although no differences were found within the experimental groups in the expression of *Tnfsf4* and *Irf4* in the MLNs (not shown), Th2 immunity was stimulated in mice that received EW + iHDM, as indicated by the expression levels of *Il4* and *Gata3* (**Figure 3A**). The assessment of the functionality of CD4^+^ T cells from the different mouse groups through the analysis of the intracellular expression of Foxp3, Interferon-γ (IFN-γ), IL-4, and IL-17 by flow cytometry, following culture of MLN cells with RPMI and EW, revealed that MLN CD4^+^ T cells from mice administered EW + iHDM significantly upregulated IL-4 after culture with EW (**Figure 3B**). Of note, cells expressing the proinflammatory cytokines IFN-γ (Th1) and IL-17 (Th17) were higher in mice receiving both EW + HDM and EW + iHDM either stimulated with EW or not. Taken together, these results show that proteinase activity is not the only determinant of the adjuvant properties of HDM, as the inactivated extract preferentially promoted Th2 responses in intestinal lymphoid tissues over its proteolytically active counterpart and induced the production of EW-specific antibodies.

## 4 Discussion

The results presented in this report show that the ability of ubiquitous HDM allergens to activate inflammatory pathways and Th2-immunity stimulates sensitization to unrelated egg allergens through the gastrointestinal route. We previously found that either HDM or iHDM acts as an adjuvant favoring airway sensitization and systemic allergy to EW and that the presence of active proteases slightly increased the adjuvant effect (22). However, unlike sensitization through the respiratory tract, the presence of active proteinases was not required for the adjuvant activity of HDM and even provided protection against sensitization in the gastrointestinal tract. Therefore, present findings reinforce the concept that the relevance of protease activity for the induction of an IgE response depends on the context in which the allergen is presented (23).

Following intragastric administration, EW alone failed to induce sensitization in agreement with previous findings (18). It is important to emphasize that, although it is well established that the oral route is not inherently sensitizing in mice, the evaluation of the influence of HDM and its inactive counterpart in the induction of allergic sensitization to EW required an *in vivo* model without the potentially biasing influence of an exogenous adjuvant. As a consequence, the immunological changes observed were modest. Nonetheless, the results unequivocally demonstrated that HDM, and particularly iHDM, exhibited adjuvant properties. This is evident as oral administration of EW in combination with the latter induced detectable EW-specific IgE and IgG1 antibodies, along with the release of MCPT-1 following challenge with EW.

The influence of HDM was pronounced in the small intestine, where it affected, to a greater extent than iHDM, the gene expression of tight junction proteins and alarmins, as well as the expression of CD86 on DCs. HDM extracts have been shown to degrade occludin and Zonula Occludens-1 (ZO-1) in the Caco-2 cell line and in human colonic biopsies, resulting in damage to the gut epithelial layer (7). Additionally, Der p 1, through its protease activity, modulates DC functions to enhance Th2 responses (24, 25). HDM proteases also indirectly contribute to the activation of ILC2s, Th2 cells, DCs, eosinophils, basophils, and mast cells through proteolytic maturation of IL-33, which enhances its bioactivity (3). In fact, in a previous study, we found that the short-term administration of HDM, as compared with iHDM, distinctively promoted the expression of genes that code for tight junction proteins, alarmins, proinflammatory, and Th2 cytokines in the small intestine, activating ILC2s and upregulating DC genes associated to Th2 polarizing properties (15). The comparatively milder intestinal effects of HDM found in the present, longer-term study suggest that proteolytic degradation of tight junctions and its consequences can be reverted through homeostatic tissue repair mechanisms (26).

Notably, the administration of EW + iHDM induced a more robust Th2 response in the LP and, particularly, in the MLNs and resulted in the production of EW-specific Th2-driven antibodies. This suggests a potential role in facilitating the delivery of antigens to lymphoid organs. Enhanced absorption of allergens through intestinal epithelial cells can promote their systemic dissemination and support oral tolerance, whereas preferential uptake through Peyer’s patches and subsequent transport to the draining MLNs is believed to enhance their ability to induce Th2 immunity and sensitizing capacity as compared to epithelial transport (18, 27, 28). In fact, our findings indicate that EW + iHDM, compared to EW + HDM, had a potentially disruptive effect on the development of tolerance that accompanies the oral administration of EW, as evidenced by a decreased expression of Foxp3 in the jejunum, although this effect was not observed in the MLNs. In contrast to our findings, Rekima et al. reported that early oral exposure of mice to proteolytically active *D. pteronyssinus* through breast milk increases the risk of allergy induced by intraperitoneal immunization to ovalbumin. However, the different experimental approach, including the age of the mice, route, and timing of sensitization, precludes a direct comparison with our study (29).

In conclusion, when acting through the gastrointestinal tract, iHDM exerted a significant influence on the development of allergic responses to EW, whereas HDM tended to promote a tolerogenic response. These findings provide evidence for the ability of ubiquitous HDM allergens to enhance the immune response to unrelated egg allergens and highlight the complex and context-dependent nature of the adjuvant activity of HDM.

## Data Availability

The datasets presented in this study can be found in online repositories. The names of the repository/repositories and accession number(s) can be found below: https://archive.org/details/osf-registrations-kyxwq-v1, 10.17605/OSF.IO/KYXWQ.

## Supplementary material

Supplementary Figure 1 Protease activity of the proteolytically active and inactive extracts assessed using the fluorogenic peptide substrate Boc-Gln-Ala-Arg-AMC.
