# Supplementary figures and images for: Ingested house dust mite favors sensitization to egg white in mice independently of its proteinase activity
Source: Front Immunol. 2025 Jan 20;15:1505003. doi: 10.3389/fimmu.2024.1505003 (PMC11788175; doi:10.3389/fimmu.2024.1505003)

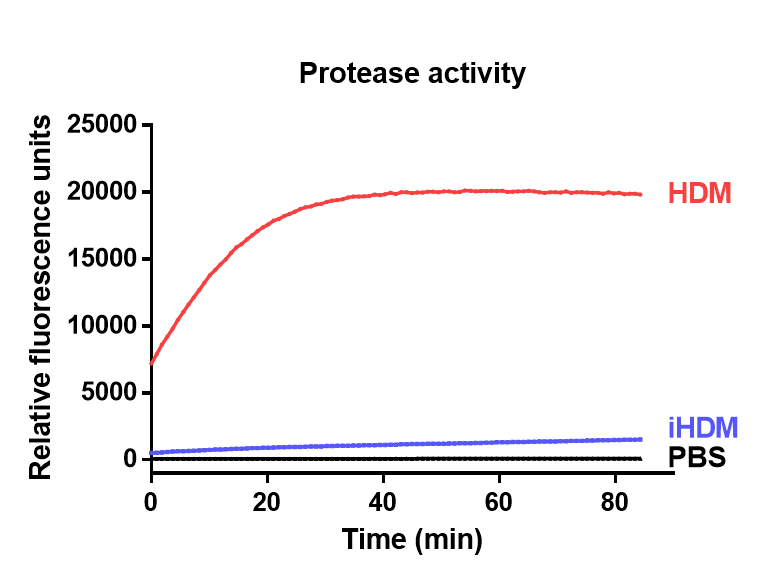

Supplement: Supplementary Figure 1 — Protease activity of the proteolytically active and inactive extracts assessed using the fluorogenic peptide substrate Boc-Gln-Ala-Arg-AMC. [file Image1.tif]
