# Supplementary material for: Ingested house dust mite favors sensitization to egg white in mice independently of its proteinase activity
Source: Front Immunol. 2025 Jan 20;15:1505003. doi: 10.3389/fimmu.2024.1505003 (PMC11788175; doi:10.3389/fimmu.2024.1505003)
Supplement: Supplementary file 2 [file Table1.docx]

| **Gene** | **Primer pairs** | **Reference** | **Cycling conditions** |
| --- | --- | --- | --- |
| *Actb* | *fw* 5' AGCTGCGTTTTACACCCTTT 3'  *rv* 5' AAGCCATGCCAATGTTGTCT 3' | Cardoso et al., 2009 | *Pre-Incubation*  *2 min 50ºC*  *Incubation*  *10 min 95ºC*  **40 cycles:**  *Denaturation*  *15 s 95ºC*  *Annealing/Extension*  45 s 58ºC + 15 s 60ºC |
| *Il6* | *fw* 5' TTCCATCCAGTTGCCTTCTTG 3'  *rv* 5' GGGAGTGGTATCCTCTGTGAAGTC 3' | Tordesillas et al., 2014 |  |
| *Il4* | *fw* 5' CCTCACAGCAACGAAGAACA 3'  *rv* 5' ATCGAAAAGCCCGAAAGAGT 3' | Yang et al., 2009 |  |
| *Il13* | *fw* 5' CATGGCCTCTGTAACCGCAA 3'  *rv* 5' CCTCATTAGAAGGGGCCGTG 3' | Pérez-Rodriguez et al., 2020 |  |
| *Jam1* | *fw 5' TTGGCGTCTGGTTTGCCTAT 3'*  *rv 5' TAGAGTAGCTGGCACCCCAT 3'* | This work |  |
| *Actb* | *fw* 5' AGCTGCGTTTTACACCCTTT 3'  *rv* 5' AAGCCATGCCAATGTTGTCT 3' | Cardoso et al., 2009 | *Pre-Incubation*  *2 min 50ºC*  *Incubation*  *10 min 95ºC*  **40 cycles**:  *Denaturation*  *15 s 95ºC*  *Annealing/Extension*  *60 s 60ºC* |
| *Il9* | *fw* 5' GTCCGTCCTTTTCCTGCGAA 3'  *rv* 5' TCTGTCTTCATGGTCGGCTT 3' | Benedé et al., 2021 |  |
| *Il33* | *fw* 5' ATTTCCCCGGCAAAGTTCAG 3'  *rv* 5' AACGGAGTCTCATGCAGTAGA 3' | Li et al., 2013 |  |
| *Il25* | *fw* 5' ACAGGGACTTGAATCGGGTC 3'  *rv* 5' TGGTAAAGTGGGACGGAGTTG 3' | Li et al., 2013 |  |
| *Tslp* | *fw* 5' AGGCTACCCTGAAACTGAGA 3'  *rv* 5' GGAGATTGCATGAAGGAATAC 3' | Negishi et al., 2012 |  |
| *Il17* | *fw 5' TGCCTGTGGCACTGAAGTAG 3'*  *rv 5' TTCATGGCTGCAGTGAAAAG 3'* | Lenoir y col., 2016 |  |
| *Gata3* | *fw* 5' CCTTAAAACTCTTGGCGTCC 3'  *rv* 5' AGACACATGTCATCCCTGAG 3' | Zhang et al., 2013 |  |
| *Foxp3* | *fw 5' ACAACCTGAGCCTGCACAAGT 3'*  *rv 5' GCCCACCTTTTCTTGGTTTTG 3'* | Cardoso y col., 2009 |  |
| *Irf4* | *fw* 5' TCCTCGTCCCTTGCTGAAAC 3'  *rv* 5' GGGCTTTGGGGCTTCTAGTT 3' | Pérez-Rodriguez et al., 2020 |  |
| *Actb* | *fw* 5' AGCTGCGTTTTACACCCTTT 3'  *rv* 5' AAGCCATGCCAATGTTGTCT 3' | Cardoso et al., 2009 | *Pre-Incubation: 2 min 50ºC*  *Incubation: 10 min 95ºC*  **40 cycles:**  *Denaturation*  *15 s 95ºC*  *Annealing/Extension*  *30 s 56ºC + 30 s 58ºC* |
| *Tjp2* | *fw 5' TGGGACCGTCGCTTTCTG 3'*  *rv 5' CTGTGGCGGGGAGGTTTGA 3'* | Benedé et al., 2021 |  |
| *Tnfsf4* | *fw* 5' GGGATGCTTCTGTGCTTCATCT 3'  *rv* 5' TTTGGATTGGAGGGTCCTTTG 3' | Mehta et al., 2016 |  |

***Supplementary Table 1:*** *Primer pair sequences for the analyses of gene expression.*

*fw, forward; rv, reverse*

**References**

Cardoso CR, Provinciatto PR, Godoi DF, Ferreira BR, Teixeira G, Rossi MA. IL-4 regulates susceptibility to intestinal inflammation in murine food allergy. Am J Physiol Gastrointest Liver Physiol. 2009;296(3):593-600. doi: 10.1152/ajpgi.90431.2008.

Tordesillas L, Goswami R, Benedé S, Grishina G, Dunkin D, Järvinen KM, Maleki SJ, Sampson HA, Berin MC. Skin exposure promotes a Th2-dependent sensitization to peanut allergens. J Clin Invest. 2014;124(11):4965-4975. doi: 10.1172/JCI75660.

Yang M, Yang C, Nau F, Pasco M, Juneja LR, Okubo T, Mine Y. Immunomodulatory effects of egg white enzymatic hydrolysates containing immunodominant epitopes in a Balb/c mouse model of egg allergy. J Agric Food Chem. 2009;57(6):2241-2248. doi: 10.1021/jf803372b. doi: 10.1021/jf803372b.

Pérez-Rodríguez L, Martínez-Blanco M, Lozano-Ojalvo D. Molina E, López-Fandiño R. Egg yolk augments type 2 immunity by activating innate cells. Eur J Nutr. 2020;59(7):3245-3256. doi: 10.1007/s00394-019-02163-6.

Benedé S, Pérez-Rodríguez L, Martínez-Blanco M, Molina E, López-Fandiño R. Oral exposure to house dust mite activates intestinal innate immunity. Foods. 2021;10(3):561. doi: 10.3390/foods10030561.

Li J, Wang Y, Tang L, Villiers WJ, Cohen D, Woodward J, Finkelman FD, Eckhardt ER. Dietary medium-chain triglycerides promote oral allergic sensitization and orally induced anaphylaxis to peanut protein in mice. J Allergy Clin Immunol. 2013;131(2):442-450. doi: 10.1016/j.jaci.2012.10.011.

Negishi H, Miki S Sarashina H, Taguchi-Atarashi N, Nakajima A, Matsuki K, Endo N, Yanai H, Nishio J, Honda K, Taniguchi T. Essential contribution of IRF3 to intestinal homeostasis and microbiota-mediated Tslp gene induction. Proc Natl Acad Sci U S A. 2012;109(51): 21016-21021. doi: 10.1073/pnas.1219482110.

Lenoir M, Del Carmen S, Cortes-Perez NG, Lozano-Ojalvo D, Muñoz-Provencio D, Chain F, Langella P, de Moreno de LeBlanc A, LeBlanc JG, Bermúdez-Humarán LG. Lactobacillus casei BL23 regulates Treg and Th17 T-cell populations and reduces DMH-associated colorectal cancer. The Journal of Gastroenterology. 2016;51(9):862-73. doi: 10.1007/s00535-015-1158-9.

Zhang C, Gui L, Xu Y, Wu T, Liu D. Preventive effects of andrographolide on the development of diabetes in autoimmune diabetic NOD mice by inducing immune tolerance. Int Immunopharmacol. 2013;16(4):451-456. doi: 10.1016/j.intimp.2013.05.002.

Mehta AK, Duan W, Doerner AM, Traves SL, Broide DH, Proud D, Zuraw BL, Croft M. Rhinovirus infection interferes with induction of tolerance to aeroantigens through OX40 ligand, thymic stromal lymphopoietin, and IL-33. J Allergy Clin Immunol. 2016;137(1):278-288. doi: 10.1016/j.jaci.2015.05.007.
